# Analysis of Plasma-Derived Exosomal MicroRNAs as Potential Biomarkers for Canine Idiopathic Epilepsy

**DOI:** 10.3390/ani14020252

**Published:** 2024-01-13

**Authors:** Mireya García-Gracia, Laura Moreno-Martinez, Adelaida Hernaiz, Sebastián Usón, Jon Moral, David Sanz-Rubio, Pilar Zaragoza, Jorge Palacio, Belén Rosado, Rosario Osta, Sylvia García-Belenguer, Inmaculada Martín Burriel

**Affiliations:** 1 Laboratorio de Genética Bioquímica (LAGENBIO), Facultad de Veterinaria, Universidad de Zaragoza, 50013 Zaragoza, Spainlauramm@unizar.es (L.M.-M.); ahernaiz@unizar.es (A.H.); pilarzar@unizar.es (P.Z.); osta@unizar.es (R.O.); 2 Instituto Agroalimentario de Aragón IA2 (UNIZAR-CITA), 50013 Zaragoza, Spain; 3 Instituto de Investigación Sanitaria Aragón (IISA), 50018 Zaragoza, Spain; 4 Centro de Investigación Biomédica en Red Enfermedades Neurodegenerativas (CIBERNED), Instituto de Salud Carlos III, 28029 Madrid, Spain; 5 Departamento de Patología Animal, Facultad de Veterinaria, Universidad de Zaragoza, Miguel Servet, 177, 50013 Zaragoza, Spainjpalacio@unizar.es (J.P.); belen@unizar.es (B.R.); sgarcia@unizar.es (S.G.-B.); 6 Hospital Veterinario de la Universidad de Zaragoza (HVUZ), 50013 Zaragoza, Spain; 7 Precision Medicine in Respiratory Diseases (PRES) Group, Unidad de Investigación Traslacional, Instituto de Investigación Sanitaria de Aragón-IISA, Hospital Universitario Miguel Servet, 50009 Zaragoza, Spain; dsanz@iisaragon.es

**Keywords:** dog, idiopathic epilepsy, microRNAs, diagnosis, exosome

## Abstract

**Simple Summary:**

Predicting drug resistance in human and canine patients with epilepsy is a feature yet to be achieved that can potentially reduce mortality. Exosomes are small extracellular vesicles that can be released by cells into the blood and are considered a source of biomarkers. Among the different existing biomarkers, microRNAs (miRNAs) appear as useful molecules in multiple diseases. In this study, miRNAs were isolated from the plasma-circulating exosomes of healthy dogs and dogs with idiopathic epilepsy. Seven potential miRNA biomarkers were analyzed. Among them, we established four prognostic biomarker candidates for drug-resistant epilepsy in dogs, linked to epileptogenesis, and four candidates for diagnostic biomarkers for epilepsy. Some of these findings exhibited similarities between the human and canine species. We propose plasma-circulating exosomes as an advantageous source of biomarkers for epilepsy-related research.

**Abstract:**

Epilepsy is one of the most prevalent complex neurological diseases in both the canine and human species, with the idiopathic form as its most common diagnosis. MicroRNAs (miRNAs) are small, noncoding RNA molecules that play a role in gene regulation processes and appear to be a promising biological target for convulsion control. These molecules have been reported as constituents of the internal content of exosomes, which are small extracellular vesicles released by cells. In this study, exosome samples were isolated from the plasma of 23 dogs, including 9 dogs with epilepsy responsive to treatment, 6 dogs with drug-resistant epilepsy, and 8 control dogs. Plasma exosomes were then characterized by electron transmission microscopy, nanoparticle tracking analysis, and dot blotting. Afterwards, the microRNA-enriched RNA content of exosomes was isolated, and miRNA quantification was performed by quantitative real-time PCR. Seven circulating miRNAs that have been previously described in the literature as potential diagnostic or prognostic biomarkers for epilepsy were evaluated. We observed significant differences in miR-16 (*p* < 0.001), miR-93-5p (*p* < 0.001), miR-142 (*p* < 0.001), miR-574 (*p* < 0.01), and miR-27 (*p* < 0.05) levels in dogs with refractory epilepsy compared to the control group. In drug-sensitive epileptic dogs, miR-142 (*p* < 0.01) showed significant differences compared to healthy dogs. Moreover, distinct levels of miR-16 (*p* < 0.05), miR-93-5p (*p* < 0.01), miR-132 (*p* < 0.05), and miR-574 (*p* < 0.05) were also found between drug-sensitive and drug-resistant epileptic dogs. Our results present plasma-circulating exosomes as an advantageous source of epileptic biomarkers, highlighting the potential of miRNAs as prognostic and diagnostic biomarkers of canine idiopathic epilepsy.

## 1. Introduction

Epilepsy is a complex neurological disease characterized by an enduring predisposition to generate epileptic seizures, which are manifestations of excessive synchronous neural activity, causing transient behavioral changes [1,2,3]. In neuroscience, the domestic dog (*Canis lupus familiaris*) is emerging as a promising natural animal model for studying cognitive, socialization, and nervous system diseases [4]. This is primarily due to the species’ cooperation and training ability, which enable researchers to conduct studies in a minimally invasive and ethical manner [4,5]. In particular, the dog is considered a relevant animal model for the study of epilepsy in humans due to the readily available information about the species’ diseases and phenotype as well as the epidemiological, clinical, and pharmacological similarities [1]. Resistance to treatment is a major issue in both dogs and humans [1], with between 30 and 40% of epileptic patients exhibiting resistance to current available treatments [6,7]. This poses a significant health risk, as untreated cases lead to increased seizure frequency and intensity, resulting in permanent damage and even premature death [8]. Therefore, determining the existence and potential causes of this resistance is essential for improving treatment outcomes in both species.

Exosomes are a subtype of extracellular vesicles that exhibit unique contents based on the sorting mechanism, determined by both the cell type of origin and the physiological or pathological status of the patients. This variability makes exosomes a subject of extensive research as a source of biomarkers, given their significant biological functions and easy accessibility from different biological fluids [9,10]. Notably, exosomes possess the ability to traverse the blood–brain barrier, enabling them to transport molecules from different parts of the body to the central nervous system [11]. Exosomes can carry diverse cargo, including proteins [10,12], lipids [10,13], and nucleic acids [10]. Particularly noteworthy is the presence of microRNA (miRNA) molecules, which are small noncoding RNAs that play a crucial role in regulating gene expression.

Evidence in the literature suggests that miRNA cargo within exosomes may serve as both a local and long-distance intercellular communication mechanism [14,15]. These noncoding RNAs could play a pivotal role in gene regulation during the development of status epilepticus and serve as biological targets for controlling seizures [14,16]. Nevertheless, miRNA dysregulation in epilepsy remains largely understudied.

Recent studies suggest that extracellular vesicles, including exosomes, could be released after brain injury or stimulation and may serve as epilepsy biomarkers. However, these studies have primarily focused on murine models with induced epilepsy rather than models with naturally occurring epilepsy [17]. Therefore, in this study, we aimed to evaluate the exosomal miRNA cargo of epileptic dogs with different treatment responses to assess their role in the disease process and to identify potential diagnostic and prognostic biomarkers for canine idiopathic epilepsy (IE).

## 2. Materials and Methods

### 2.1. Patient Recruitment, Sample Collection, and Exosome Characterization

#### 2.1.1. Animals and Procedures

Dogs were recruited in the neurology service of the Veterinary Hospital of the University of Zaragoza (*n* = 23). The group of epileptic dogs was made up of patients diagnosed with IE according to at least the Tier I confidence level criteria from the International Veterinary Epilepsy Task Force (IVETF) [18]. Epileptic dogs were classified as DSE (drug-sensitive epilepsy) when they were being treated with one single antiepileptic drug with a good clinical response (*n* = 9) or DRE (drug-refractory epilepsy) when they were under multidrug treatment without achieving sustained seizure freedom after at least two appropriate antiseizure drug trials (*n* = 6). None of these dogs suffered from any other pathology except IE (Supplementary Table S1).

The control group (*n* = 8) was made up of healthy dogs that were as closely matched as possible in terms of breed, sex, and age with the epileptic group (Supplementary Table S1).

Blood samples with a volume of approximately 2 mL were obtained by jugular venipuncture from both epileptic and control dogs. Immediately after collection, blood samples were centrifuged at 1300× *g* for 10 min, and the resulting plasma fraction was stored at −80 °C until needed.

Written informed consent was obtained from owners for the participation of their dogs in this study. They were given the opportunity to ask any questions and confirm or decline participation. All procedures were carried out under Project License PI67/21, approved by the Ethics Committee for Animal Experimentation of the University of Zaragoza.

#### 2.1.2. Exosome Purification from Plasma

Exosome isolation was performed using 300 μL of plasma fractions with the miRCURY^®^ Exosome Serum/Plasma kit (QIAGEN, Valencia, CA, USA). Thawed plasma samples were initially centrifuged at 3000× *g* for 10 min to remove cells and debris before proceeding with the recommended protocol. This protocol consisted of the addition of a precipitation buffer, which diminishes the hydration of the subcellular particles and allows precipitation of small particles with a low-speed centrifugation step, followed by the incubation of the mixture for 1 h at 4 °C. Subsequently, the sample was centrifuged at 500× *g* for 5 min to obtain the extracellular vesicle pellet.

For exosome characterization, we utilized plasma samples from 3 controls and 5 epileptic dogs, of which 3 were drug-sensitive (DSE) dogs and 2 were drug-refractory (DRE) dogs. (Supplementary Table S1). The protocol was applied twice to each sample. One of the exosome pellets was resuspended in 100 μL of filtered PBS, from which a 50 μL aliquot was set aside for nano tracking analysis (NTA), and various dilutions were prepared with the remaining volume to optimize transmission electron microscopy (TEM) observation. The other pellet was resuspended in 100 μL of RIPA buffer for further protein marker analysis. All aliquots were stored at −20 °C until needed.

#### 2.1.3. Determination of Exosome Markers by Dot Blotting

The presence of exosomes in the recovered pellet was confirmed by analyzing the presence of exosome markers. The Exo-Check antibody array (System Biosciences, Palo Alto, CA, USA) was used for this purpose. Each array consists of a membrane with 12 preprinted spots and features 8 antibodies against known human exosome markers (CD63, CD81, ALIX, FLOT1, ICAM1, EpCam, ANXA5, and TSG101) and the cis-Golgi marker CM130, used to monitor cellular contamination. We followed the manufacturer’s instructions. For this analysis, 10 μL of exosome isolates diluted in RIPA buffer were further diluted in a 1:4 ratio in the same buffer. Protein lysates were hybridized with the preprinted spots, and a signal was developed using a secondary detection mixture conjugated to HRP. Chemiluminescence signals were detected in a ChemiDoc Imaging System (Biorad, Hercules, CA, USA).

#### 2.1.4. Imaging of Exosomes Using Transmission Electron Microscopy

Exosome samples diluted in PBS were further diluted at ratios of 1:50, 1:100, 1:200, and 1:1000 using filtered PBS to determine the optimal dilution for exosome visualization. Subsequently, the preparations were negatively stained with 2% uracil acetate in water. The staining and sample handling were performed at the Electronic Microscopy of Biological Systems Service of the University of Zaragoza. To visualize exosome morphology, the Tecnai T20 microscope (Thermo Fisher Scientific, Waltham, MA, USA) was employed at the Materials Electronic Microscopy Service of the University of Zaragoza. Images were captured using the Veleta CCD 2K × 2K high-speed microscope camera (Olympus, Shinjuku, Tokyo, Japan) with a working voltage of 200 kV.

#### 2.1.5. Analysis of Exosome Size and Concentration Using Nano Tracking Analysis

Particle size and concentration were determined using the NanoSight NS300 (Malvern Panalytical, Malvern, UK) that allows rapid and automatic analysis of the size distribution and concentration of all types of nanoparticles, from 10 to 1000 nm in diameter and, depending on the sample, from 10^6^ to 10^9^ particles/mL. To minimize the potential source of error, for each dog, the same volume of plasma (300 μL) was used for exosome isolation, and pellets were diluted in the same volume of PBS (100 μL). Exosome samples were further diluted 1000-fold with filtered PBS and EDTA (50 nM) to a final volume of 1 mL. Sets of five one-minute videos were recorded and automatically analyzed using the NTA 3.0 software (with settings of 2–4 screen gain, 11 camera level, and 5 threshold) for each sample at an operating temperature of 23 °C.

### 2.2. Exosomal miRNA Analysis

#### 2.2.1. RNA Extraction

MiRNA expression profiles were determined in a total cohort of 23 dogs (Supplementary Table S1). Initially, exosomes were isolated from 200 μL plasma samples using the miRCURY^®^ Exosome Serum/Plasma kit (QIAGEN, Valencia, CA, USA). The resulting pellet was resuspended in 200 μL of the kit’s resuspension buffer. Immediately after exosome isolation, the dilution was centrifuged for 2 min at 400× *g*, and RNA extraction was carried out using the miRNeasy serum/plasma kit (QIAGEN, Valencia, CA, USA), following the manufacturer’s instructions. After the addition of the QIAzol Lysis Reagent (QIAGEN, Valencia, CA, USA), a volume of 5.7 μL of the exogenous control cel-miR-39 (QIAGEN, Valencia, CA, USA) was pipetted into each sample. After elution, the samples were stored at −80 °C until further use.

#### 2.2.2. Reverse Transcription and Quantitative Real-Time Polymerase Chain Reaction (RT-qPCR)

A set of 7 microRNAs was selected from the bibliography due to their dysregulation in plasma in different types of human epilepsy and in mouse and rat models of human temporal lobe epilepsy [19,20,21,22,23] (Supplementary Table S2). In addition, the exogenous miRNAs cel-miR-39-3p and miR-146a were analyzed as exogenous and potential endogenous references, respectively. A primer pool was created by combining equal volumes (9 μL) of the nine specific miRNA primers (Supplementary Table S2). Transcription to cDNA was carried out with miScript Reverse Transcriptase Mix (QIAGEN, Valencia, CA, USA) using 3 μL of total RNA. Taqman primers were employed for reverse transcription on a standard thermocycler (2720 Thermal Cycler, Applied Biosystems, Waltham, MA, USA), incubating samples for 30 min at 16 °C, 30 min at 42 °C, 30 min at 85 °C, and finally holding them at 4 °C until retrieval. The resulting cDNA was stored at −80 °C.

To quantify exosomal miRNA levels, cDNA was diluted 1:5 prior to quantitative real-time PCR analysis. An amplification mix was then prepared for each miRNA, consisting of TaqMan Fast Universal PCR Master Mix (2×) (Thermo Fisher Scientific, Waltham, MA, USA) and the corresponding miRNA assay (20×) (Thermo Fisher Scientific, Waltham, MA, USA). Each RT-qPCR reaction had a total volume of 5 μL, containing 2.75 μL of the amplification mix and 2.25 μL of cDNA. To ensure accuracy, a negative control was included on each plate, and each sample was analyzed in triplicate. The following 40-cycle program was run on the Step OneTM thermocycler (Thermo Fisher Scientific, Waltham, MA, USA): 20 s at 95 °C, 1 s at 95 °C, and 20 s at 60 °C.

### 2.3. Statistical Analysis

To assess changes in miRNA expression levels, we employed the 2^-^∆∆Ct method [24]. The stability of the nine canine miRNAs in the cohort was determined using the online tool RefFinder [25,26], which identified miR-146a as the most stable miRNA, serving as an internal control. Data normalization was then performed using miR-146a as the endogenous control and cel-miR-39 as the exogenous control for each sample [27,28]. Differences between groups were evaluated using one-way analysis of variance (ANOVA), Tukey’s multiple comparison, and Kruskal–Wallis tests. Significant differences were considered at *p* < 0.05. Pearson’s correlation was calculated between the gene expression data (ΔCt values) of the different microRNAs to determine any possible relationship between expression levels.

Receiver operating characteristic (ROC) curve analysis of miRNAs was conducted using GraphPad Prism Software version 10.0 for Windows, GraphPad Software (Boston, MA, USA), and IBM SPSS Statistics software version 20 (IBM Corp. Released 2011. IBM SPSS Statistics for Windows, Version 20.0. Armonk, NY, USA: IBM Corp.).

The target prediction for each miRNA was carried out using the online database miRDB [29,30]. After the identification of miRNA targets, Kyoto encyclopedia of genes and genomes (KEGG) and gene ontology (GO) enrichment analyses were performed using the online tool ShinyGO version 0.77 [31,32,33]. Enriched pathways with FDR < 0.05 were considered statistically significant.

## 3. Results

### 3.1. Exosome Characterization

#### 3.1.1. Determination of Exosomal Markers by Dot Blotting

**Figure 1:**
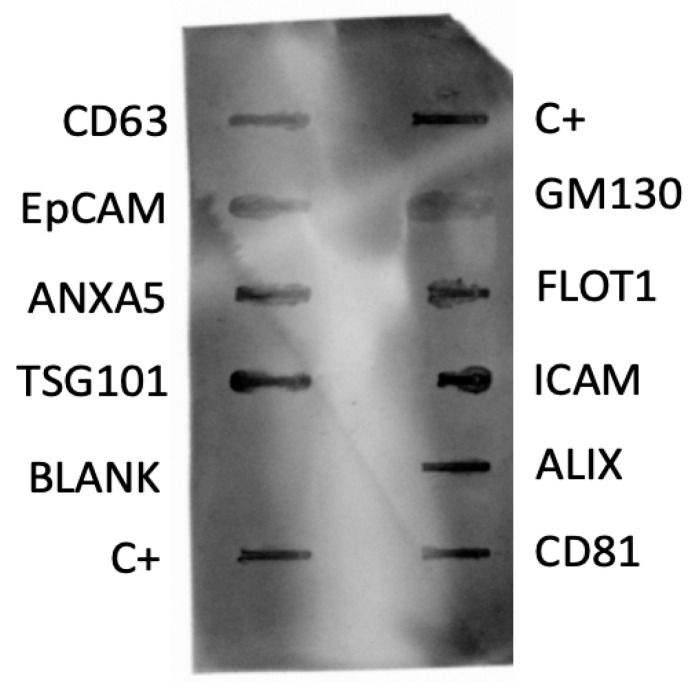
Dot blot membrane for the identification of exosome markers (CD63, EpCAM, ANXA5, TSG101, CD81, ALIX, ICAM, FLOT1) and the cis-Golgi cell contamination marker (GM130). The membrane includes two positive controls (C+) and one blank (BLANK). A plasma sample from a male refractory epileptic dog was used for this assay.

Eight exosome markers and one cis-Golgi marker were examined via dot blotting. Although the membrane antibodies target human epitopes, strong reactivity was observed for TSG101, FLOT1, ICAM, ALIX, and CD81, while a weaker signal was detected for CD63, EpCAM, and ANXA5 (Figure 1). The varied marker reactivity may be explained by differences in the affinity of the antibodies for canine epitopes. The negative result for the cis-Golgi contamination marker (GM130) dismissed the possibility of cell contamination in the sample extraction and manipulation.

#### 3.1.2. Imaging Exosomes Using Transmission Electron Microscopy (TEM)

**Figure 2:**
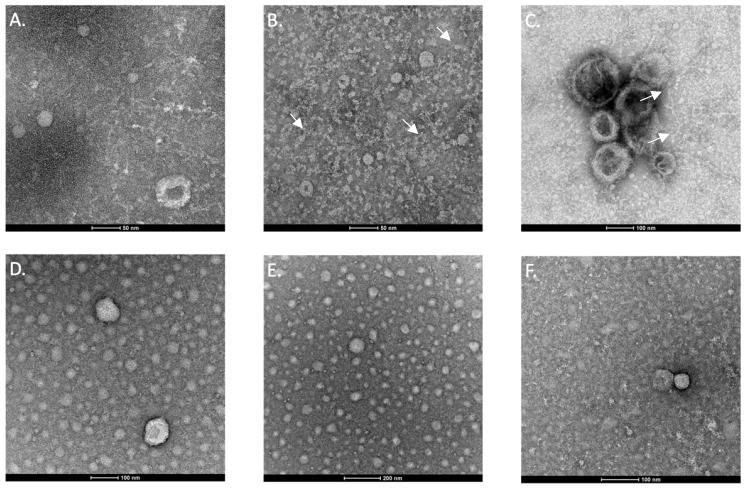
Transmission electron microscopy images of isolated exosomes. The upper row showcases images of undiluted samples obtained from a treatment-sensitive dog (**A**), a treatment-resistant dog (**B**), and a control dog (**C**). The lower row displays images of 1:200 PBS-diluted samples obtained from a drug-sensitive dog (**D**), a drug-resistant dog (**E**), and a control dog (**F**). White arrows indicate the presence of “cup-like” exosomes.

Extracellular vesicles obtained from canine plasma were subjected to analysis using TEM. While most of the vesicles observed displayed a spherical shape, some exhibited a cup-like shape due to the dehydration step in the staining method (Figure 2) [34]. We conducted various dilutions to optimize the visualization using TEM. The undiluted sample presented background noise that complicated visualization; however, the image became clearer with different dilutions. We observed that diluting the samples in a 1:200 ratio in PBS removed most of the noise and provided the best structure visualization, along with a more uniform distribution of exosomes. All samples contained vesicles within the size range typically associated with exosomes (ranging from 30 to 150 nm) [35,36,37]. Additionally, an abundance of medium-sized particles was observed, as well as smaller ones.

#### 3.1.3. Analysis of Extracellular Vesicle Size and Concentration Using Nano Tracking Analysis (NTA)

**Figure 3:**
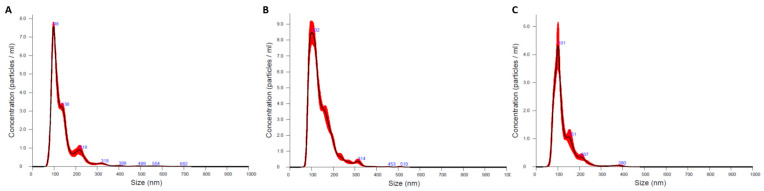
Size distribution of detected nanoparticles in dogs with drug-sensitive (**A**), drug-refractory epilepsy (**B**), and controls (**C**). The black line depicts the size distribution against particle concentration/mL, with the standard error represented by the red, shaded areas.

The NTA results (Figure 3) confirmed that most of the particles fell within the established size range for exosomes [35,36,37]. Furthermore, we observed a lower concentration of larger particles, which may be attributed to the presence of larger extracellular particles or the formation of small particle clusters.

As shown in Table 1, most particles fell within the established size range (minimum = 84.8 nm; maximum = 132.9 nm), with an average diameter of 99.8 nm. The concentration of particles was variable, with values ranging between 2.40 × 10^11^ and 7.85 × 10^11^ particles/mL. Due to the low number of animals in each group, we did not conduct statistical analysis.

### 3.2. The miRNA Analysis

#### 3.2.1. The miRNA Expression Analysis Using RT-qPCR

**Figure 4:**
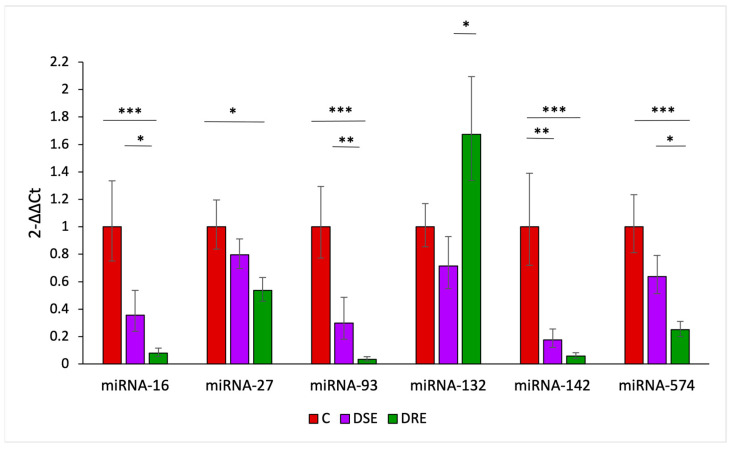
Relative changes of miR-16, miR-27, miR-93-5p, miR-132, miR-142, and miR-574 levels arranged by condition (control (C), drug-sensitive epilepsy (DSE), and drug-refractory epilepsy (DRE)). Data are presented as 2^−∆∆Ct^ mean values for each cohort ± standard error (SE). *** *p* < 0.001; ** *p* < 0.01; * *p* < 0.05.

All selected miRNAs were successfully amplified using RT-qPCR in plasma-derived exosomes isolated from dogs. Six out of the seven examined miRNAs showed significant changes in epileptic dogs (Figure 4).

MicroRNAs miR-16 (*p* < 0.001; *p* < 0.05), miR-93-5p (*p* < 0.001; *p* < 0.01), and miR-574 (*p* < 0.01; *p* < 0.05) displayed a significant downregulation in refractory epileptic dogs when compared to both control and DSE animals. In addition, miR-27 (*p* < 0.05) exhibited downregulation in DRE animals compared to the control group, while miR-142 showed a statistically significant reduction in both treatment-sensitive (*p* < 0.01) and treatment-refractory dogs (*p* < 0.001) when compared to controls. Only miR-132 displayed upregulation in refractory epileptic dogs, and this increase reached statistical significance when compared to DSE dogs (*p* < 0.05).

Several pairs of microRNAs displayed significant Pearson’s correlation values (Supplementary Table S3). Different degrees of positive and significant correlation were found between miR-16, miR-27, miR-93, miR142, and miR-574, with the highest correlation values observed between miR-93 and miR-19 (r = 0.941, *p* < 0.001) and miR-93 and miR-142 (r = 0.878, *p* < 0.001).

#### 3.2.2. Discriminative Ability Analysis

**Figure 5:**
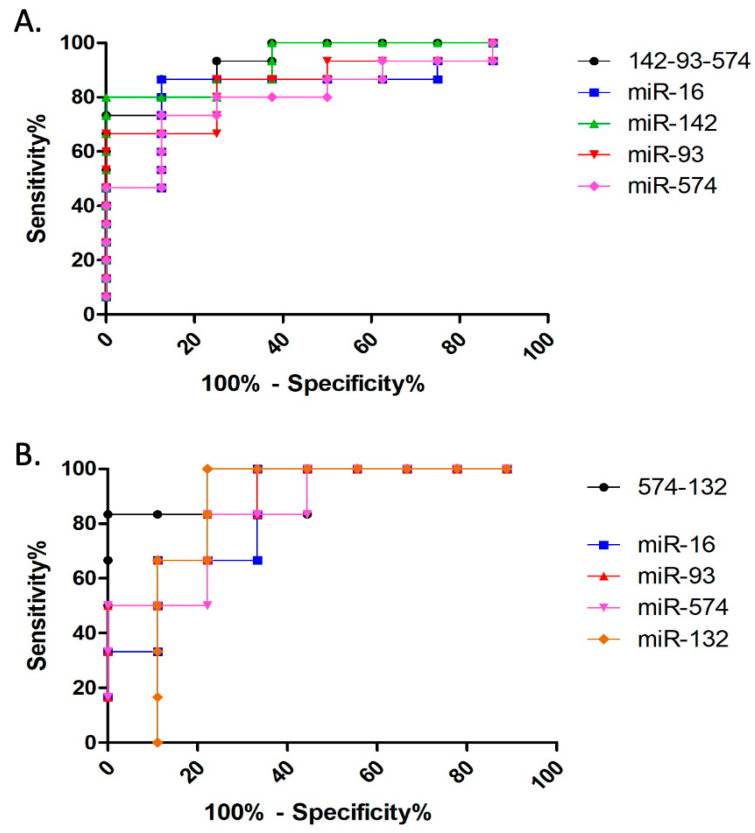
ROC curve analysis of diagnostic performance of combined miR-93-5p, miR-142, and miR-574; miR-16; miR-93-5p; miR-142; and miR-574 (**A**); and ROC curve analysis of prognostic performance of combined miR-132 and miR-574; miR-16; miR-93-5p; miR-132; and miR-574 (**B**).

An ROC curve analysis was conducted to assess the prognostic and diagnostic performance of miRNAs (Figure 5) [38]. When comparing control animals with epileptic dogs regardless of condition, exosomal miRNAs miR-16 (AUC = 0.8417; *p* = 0.0082), miR-93-5p (AUC = 0.8583; *p* = 0.0055), miR-142 (AUC = 0.9333; *p* = 0.0008), and miR-574 (AUC = 0.8167; *p* = 0.0142) demonstrated strong diagnostic potential (Figure 5A).

Moreover, when comparing DSE animals with DRE dogs, miR-16 (AUC = 0.8519; *p* = 0.0252), miR-93-5p (AUC = 0.8889; *p* = 0.0134), miR-132 (AUC = 0.8519; *p* = 0.0252), and miR-574 (AUC = 0.8519; *p* = 0. 0252) were identified as reliable prognostic biomarkers (Figure 5B).

ROC curve analysis was also carried out with different miRNA combinations to study the possibility of improvement in diagnostic and prognostic performance. All combinations presented statistical significance (Supplementary Table S4). Those combinations that displayed a better AUC and *p*-value were selected and compared with single miRNA results (Figure 5A,B). For diagnosis, combined miR-93-5p, miR-142, and miR-574 had the best performance (AUC = 0.9417; *p* = 0.0006) (Figure 5A). For prognostic performance, combined miR-132 and miR-574 showed the best results (AUC = 0.9259; *p* = 0.0067) (Figure 5B).

#### 3.2.3. Target Prediction and Enrichment Analysis

All six significantly expressed miRNAs had several associated gene targets: 200 were linked to miR-16, 261 to miR-27a-3p, 230 to miR-93-5p, 75 to miR-132, 159 to miR-142, and 1 to miR-574-3p (Supplementary Table S5). Enrichment analyses were performed for all miRNA targets, except for miR-574, which only had one associated target.

KEGG pathway analysis showed no significant pathways for miR-132 and miR-142 targets. On the other hand, miR-16 targets revealed significant enrichment in MTOR, FoxO, and PI3K-Akt signaling pathways, autophagy, cell cycle, and oocyte meiosis. The miR-27a-3p targets were only enriched in the EGFR tyrosine kinase inhibitor resistance pathway, whereas the miR-93-5p targets were associated with circadian rhythm and endocytosis (Table 2).

The GO enrichment analysis neither revealed significant enrichments for miR-142 nor miR-132 gene targets. Regarding miR-16 gene targets, they were enriched in biological processes such as regulation of protein autophosphorylation, sodium ion transport, establishment or maintenance of cell polarity, and negative regulation of lamellipodium organization, among others. These targets were also enriched in the Golgi apparatus cellular component and in several molecular functions such as proton, sodium, and cation symporter activities, active ion transmembrane transporter activity, protein kinase activity, and GTPase inhibitor activity, among others (Supplementary Table S6).

Some of the enriched biological processes for miR-27a-3p gene targets were related to neuron development, generation of neurons, neuron differentiation, neurogenesis, and regulation of synaptic plasticity. These targets were also associated with the molecular functions of nuclear receptor activity and ligand-activated transcription factor activity (Supplementary Table S6).

miR-93-5p gene targets were associated with regulation of endocytosis and regulation of vesicle-mediated transport processes (Supplementary Table S6).

## 4. Discussion

Idiopathic epilepsy is a relatively common disease, with a prevalence of 0.60–0.75% in dogs and 0.52–0.89% in humans [1,39]. In both species, a third of patients are resistant to antiseizure treatments, diminishing their quality of life and leading to a risk of premature death [5,6,7,40]. The development of prognostic biomarkers could accelerate the diagnosis of idiopathic epilepsy, and the identification of early resistance to treatment would help in making decisions regarding the prescription of alternative therapies. In this work, we have taken a first step toward the discovery of diagnostic and prognostic biomarkers for canine idiopathic epilepsy.

Exosome release has been reported in most cells, and its presence has been observed in various biological fluids [41]. Moreover, these extracellular vesicles are also known as key mediators in several neurological diseases, including epilepsy, in which alterations in exosome release and its cargo have been documented [11,17]. Within the exosome cargo of human patients, miRNAs particularly show differential expression between patients with refractory and sensitive epilepsy related to focal cortical dysplasia [42]. Given the similarities in the presentation of idiopathic epilepsy in both humans and canines, our study aimed to investigate whether circulating miRNA changes, previously observed in human patients with different forms of epilepsy [19,20,21,22,23], could also serve as potential biomarkers in the canine species, enabling us to improve the diagnostic and prognostic tools in veterinary science for this disease.

In the present work, we optimized the protocol to isolate exosomes and purify small RNA from a small volume of dog plasma. The presence of extracellular vesicles in plasma was confirmed using TEM, and the appearance of these particles was consistent with the typical morphology of exosomes. Moreover, the sizes obtained using NTA fell within the established range for exosomes [35,36,37], and the high particle concentration values implied a promising abundance of these extracellular vesicles in dog plasma. Moreover, the identification of exosomal markers confirmed the nature of the isolated vesicles. We observed immunoreactivity for CD63 and CD81, which participate in the sorting mechanism of exosomes [41]. Additionally, we detected strong reactivity for ALIX and TSG101, which are considered general exosome markers due to their ubiquity and abundance [43,44,45,46,47].

As previously mentioned, miRNAs are present in the cargo of exosomes. These molecules have great biomarker potential, particularly those contained within these extracellular vesicles, which are secreted by various cells within the organism, including those of the central nervous system, and their content is influenced by the individual’s physiological state [8,9,10]. In this study, we analyzed the levels of seven miRNAs known to be altered in human patients with epilepsy [19,20,21,22,23]. The miRNAs were isolated from plasma-derived exosomes obtained from both epileptic and control dogs, and six of the seven studied miRNAs were altered in epileptic dogs (miR-16, miR-27a-3p, miR-93-5p, miR-132, miR-142, and miR-574-3p), with the downregulation of five of them being highly correlated.

Our study revealed lower levels of miR-16 (GC13M050048) in dogs with DRE compared to dogs with DSE and controls, despite having included this microRNA in the study as a potential internal reference due to its prior use in human plasma samples [21,27]. To the best of our knowledge, no miR-16 alterations have been described in human or canine epileptic patients. However, in rat models of pilocarpine-induced epilepsy, the levels of miR-16 varied in different directions depending on the brain area [48]. In the hippocampus, the increase observed after status epilepticus was reduced with palliative treatment with paroxetine, which was linked with a modulation of apoptosis [48]. We cannot discard that the observed decrease in this and other microRNAs was a response related to treatment, as all the epileptic animals studied are on some form of antiepileptic treatment. According to the enrichment analysis, genes potentially regulated by miR-16 were enriched in autophagy and the MTOR signaling pathway, which is implicated in the cell life cycle [49]. Increasing expression levels of this miRNA have also been reported in astrocytes obtained from mice with encephalitis B-induced epilepsy [50], with this increase associated with a potential role in the regulation of inflammation processes.

Regarding miR-27a-3p (GC19M017490), the diminished levels observed in DRE dogs compared to the control ones contrast with the findings in human serum, where no significant differences in expression levels were found between control and epileptic patients [19]. However, our results are similar to the downregulation of miR-27a-3p observed in murine plasma after seizures [51]. This miRNA was also enriched in different processes related to neurogenesis, neuron differentiation, and synaptic plasticity. Interestingly, changes in neurogenesis have been linked to epileptogenesis [52]. In addition, in a mouse model of hypoxia-induced neural apoptosis, miR-27a-3p overexpression seems to inhibit neural apoptosis [53,54], and in a rat model with intracerebral hemorrhage (ICH), restoration of this miRNA seems to maintain blood–brain barrier permeability and reduce brain edema, among other processes [54,55].

In contrast with the increased levels of miR-93-5p (GC07M103953) found in plasma samples of humans and mice with epilepsy [23], this miRNA was decreased in refractory-to-treatment dogs. Brennan et al. [23] showed that dysregulation of miR-93-5p in epileptic humans and mice could be associated with the development of the disease and be useful for tracking the efficacy of novel antiepileptogenic therapies. Furthermore, they also propose this miRNA as a potential biomarker to assist in the diagnosis of epilepsy, although with certain limitations [23]. The miR-93-5p gene has also been implicated in various biological processes, including the negative regulation of brain-related cytokine production [56] and the negative regulation of protein release [56]. Gene targets linked to miR-93-5p were enriched in several functions, including endocytosis, circadian rhythm, and regulation of vesicle-mediated transport. Receptor endocytosis is a regulatory mechanism that controls cell surface expression and the subsequent contribution of selective neurotransmitter receptors toward synaptic transmission [57]. In a hippocampal culture model of epilepsy, epileptogenesis can induce acute and chronic increases in GABAA receptor endocytosis, contributing to the generation of seizures [52]. Circadian rhythm is also involved in epilepsy, regulating sleep structure, cortical excitability, and potentially seizure susceptibility [58]. Similar to miR-16, the downregulation of miR-93-5p levels in refractory dogs could be implicated in disease development and involved in treatment-resistance processes.

Circulating miR-142 (GC17M058331) was also downregulated in DSE and DRE dogs. These findings contrast with those in human patients with treatment-resistant temporal lobe epilepsy, in which circulating miR-142 is upregulated [20]. However, in a murine epilepsy model, miR-142 was downregulated in the brains of multidrug-resistant animals [59]. This miRNA is known to play a role in the neuroinflammatory response [60], the regulation of interleukin-1 alfa production [61], and the positive regulation of microglial cell activation. It has been associated with genes involved in neuroinflammation like “transforming growth factor beta receptor 2” (*TGFBR2*), “mothers against decapentaplegic homolog 3” (*SMAD3*), and genes related to pharmaco-resistance, like the one coding for the multidrug resistance protein 1 (*MDR1*) [21]. Positive regulation of TFG-beta factor has been linked to neuroinflammation at the blood–brain barrier, GABA inhibition, and increased excitatory synapsis [59]. Therefore, the decreased expression levels of miR-142 could be linked to neuroinflammation processes and affect drug transport and metabolization, suggesting the involvement of this marker in treatment resistance.

miR-574-3p (GC04P039043) levels decrease during epileptogenesis and epilepsy in murine and rat models [23]. Our results agree with these findings, as we observed reduced levels of this miRNA in treatment-resistant epileptic dogs compared to control and DSE animals. The only known reliable target of this microRNA is ATG2B, a gene related to autophagy regulation [62].

The only miRNA that exhibited higher levels in refractory epileptic dogs was miR-132 (GC17M002049), although this increment was only significant when compared to sensitive epileptic dogs. This evidence differs from a study in human serum where miR-132 experienced no significant changes in drug-resistant epileptic patients [21]. In rat models, this miRNA was upregulated during the acute seizure phase and was the only one still upregulated 24 h after status epilepticus induction in adult and infantile-onset temporal lobe epilepsy [63]. In addition, miR-132 seems to regulate dendrite growth and may contribute to actin remodeling [64].

Variations in sample characteristics compared to other studies [19,20,21,22,23], as well as the specificity of the origin of both exosomes and miRNA expression changes, can pose challenges in establishing interspecies epilepsy-related biomarker candidates and in the interpretation of their potential role in the disease. Despite these considerations, we were able to obtain statistically significant evidence linking several miRNA candidates to epilepsy and drug resistance. The combination of three miRNAs (miR-93-5p, miR-142, and miR-574) had good diagnostic performance. However, additional analyses including untreated epileptic dogs along with dogs with DSE or DRE are needed to verify the diagnostic ability of this combination of miRNAs at a very early stage of the disease.

Finally, it is important to mention certain limitations present in our study. Although our patient cohort was carefully balanced, it is important to note that the sample size was relatively small, and the cohorts displayed some heterogeneity. However, an ideal biomarker would be one capable of diagnosing or prognosing any dog, regardless of age, sex, or breed. In addition, treatment interactions must be taken into account, as they can also affect miRNA expression levels. Further research using a larger sample size, including drug-sensitive, drug-resistant, and untreated epileptic dogs, is necessary to draw more robust conclusions about the role of these miRNAs in canine idiopathic epilepsy and their potential as diagnostic and prognostic biomarkers.

## 5. Conclusions

While IE is the most common neurological disease in dogs [1], it appears that molecular biology-based research on this disorder remains relatively understudied in veterinary science. The absence of a standardized method of molecular analysis in humans and canines makes it difficult to establish robust cross-species connections as translational models. Nevertheless, we have observed dysregulation of several disease-relevant miRNAs (miR-16, miR-27, miR-93-5p, miR-142, miR-574, and miR-132) in dogs with drug-sensitive and drug-refractory IE that have also been found altered in human patients. Additionally, miR-93-5p, miR-142, and miR-574 emerged as potential diagnostic biomarker combinations, while combined miR-132 and miR-574 appeared as a promising prognostic biomarker candidate for dogs with refractory epilepsy. More studies are warranted to explore the exact functions of these miRNAs and their ability to act as biomarkers of canine IE.

## Figures and Tables

**Table 1:** Average diameter of particles (mode ± standard error) and particle concentration (mode ± standard error) obtained from NanoSight analysis in dogs with treatment-refractory (DRE), treatment-sensitive epilepsy (DSE), and controls (C).

Condition	Average Diameter of Particle (nm)	Concentration (Particle/mL)
DRE1	104 ± 4.4	6.81 × 10^11^ ± 6.93 × 10^9^
DRE3	102 ± 4.9	7.85 × 10^11^ ± 3.43 × 10^10^
DSE2	89.4 ± 5.5	6.94 × 10^10^ ± 8.45 × 10^9^
DSE5	89.3 ± 3.4	7.60 × 10^11^ ± 9.73 × 10^9^
DSE10	84.8 ± 2.3	4.57 × 10^11^ ± 1.77 × 10^10^
C9	98.4 ± 1.2	4.31 × 10^11^ ± 3.02 × 10^9^
C10	97.5 ± 3.9	2.40 × 10^11^ ± 6.09 × 10^9^
C11	132.9 ± 3.4	7.61 × 10^11^ ± 1.84 × 10^10^

**Table 2:** KEGG pathways enriched in miR-16, miR-27a-3p, and miR-93-5p gene targets, showing the number of genes per pathway (nGenes), the fold enrichment, and the FDR of each pathway.

miRNA	Pathway	nGenes	Fold Enrichment	FDR
miR-16	Oocyte meiosis	7	6.8	1.7 × 10^−2^
	Cell cycle	6	5.9	2.8 × 10^−2^
	FoxO signaling pathway	6	5.7	2.8 × 10^−2^
	MTOR signaling pathway	7	5.7	2.6 × 10^−2^
	Autophagy	6	5.3	3.4 × 10^−2^
	PI3K-Akt signaling pathway	10	3.5	2.8 × 10^−2^
miR-27a-3p	EGFR tyrosine kinase inhibitor resistance	6	7.1	4.8 × 10^−2^
miR-93-5p	Circadian rhythm	4	13.7	1.9 × 10^−2^
	Endocytosis	13	5.5	1.7 × 10^−4^

## Data Availability

The data presented in this study are available within the article and the provided Supplementary Materials. Real-time PCR Ct data for each microRNA are available at Zenodo.org, along with https://doi.org/10.5281/zenodo.10459397 (accessed on 4 January 2024).

## Supplementary Materials

The following supporting information can be downloaded at: https://www.mdpi.com/article/10.3390/ani14020252/s1, Table S1: Characteristics of controls and dogs with refractory idiopathic epilepsy (DRE) and sensible idiopathic epilepsy (DSE) analyzed in this study and their treatments (Pb = phenobarbital; BrK = potassium bromide); Table S2: Set of miRNAs analyzed: reference in the miRbase, mature nucleotide sequence, and reference for their selection; Table S3: Pearson’s correlation between microRNA expression data. Statistical significance: *** *p* < 0.001, ** *p* < 0.01, * *p* < 0.05; Table S4: Data from combined miRNA ROC curve analysis. Statistical significance: *** *p* < 0.001, ** *p* < 0.01, * *p* < 0.05; Table S5: Set of statistically significant miRNAs’ predicted targets on miRDB; Table S6: GO pathways in terms of biological process (BP), cellular component (CC), and molecular function (MF) enriched in miR-16, miR-27a-3p, and miR-93-5p gene targets, showing the number of genes per pathway (nGenes), the fold enrichment, and the FDR of each pathway.
