# Supplementary material for: Analysis of Plasma-Derived Exosomal MicroRNAs as Potential Biomarkers for Canine Idiopathic Epilepsy
Source: Animals (Basel). 2024 Jan 13;14(2):252. doi: 10.3390/ani14020252 (PMC10812621; doi:10.3390/ani14020252)
Supplement: Supplementary file 1 [file animals-14-00252-s001.zip › Supplementary_Files_4-2_rev.pdf]

Table S1. Characteristics of controls and dogs with Drug-Resistant Epilepsy (DRE), Drug-Sensitive Epilepsy (DSE) and controls (C) analyzed in this study, their treatments (Pb = phenobarbital; BrK = potassium bromide) and their use in the study (microRNA quantification, miRNA, or Extracellular vesicle characterization, EVC).

Condition	Age (yrs)	Sex	Reproductive status	Breed	Treatment	Assay
C1	5	Male	Neutered	Staffordshire	-	miRNA
C2	9	Female	Neutered	Labrador	-	miRNA
C3	7	Female	Neutered	Staffordshire	-	miRNA
C4	6	Male	Not neutered	Mix	-	miRNA
C5	5	Male	Neutered	Border Collie	-	miRNA
C6	2	Female	Not neutered	Border Collie	-	miRNA
C7	7	Male	Neutered	Labrador	-	miRNA
C8	6	Female	Not neutered	White Swiss Shepherd	-	miRNA
C9	12	Female	Neutered	Staffordshire	-	EVC
C10	9	Female	Neutered	Border Collie		EVC
C11	8	Male	Neutered	Border Collie	-	EVC
DRE1	5	Female	Neutered	Mastiff	Pb + Imepitoin + Levetiracetam	miRNA,EVC
DRE2	10	Female	Neutered	Labrador mix	Pb + BrK + Levetiracetam	miRNA
DRE3	5	Female	Neutered	Border Collie	Pb + BrK + Levetiracetam	miRNA, EVC
DRE4	2	Male	Not neutered	Border Collie	Pb, Imepitoin, Levetiracetam, BrK	miRNA
DRE5	10	Male	Not neutered	Bichon	Levetiracetam, BrK	miRNA
DRE6	6	Male	Neutered	Mastiff	Pb + Levetiracetam	miRNA
DSE1	2	Male	Neutered	Beagle	Pb	miRNA
DSE2	9	Male	Not neutered	Cocker	Levetiracetam	miRNA, ECV
DSE3	11	Female	Neutered	Mix	Levetiracetam	miRNA
DSE4	3	Male	Neutered	Corgi mix	Pb	miRNA
DSE5	13	Female	Not neutered	Podenco mix	Imepitoin	miRNA, EVC
DSE6	11	Male	Not neutered	Labrador	Pb	miRNA
DSE7	9	Male	Not neutered	Podenco mix	Pb	miRNA
DSE8	8	Female	Neutered	Mix	Pb	miRNA
DSE9	9	Female	Neutered	Mix	Imepitoin	miRNA
DSE10	6	Male	Not neutered	Border Collie	Pb	EVC

Table S2. Set of miRNAs analyzed: reference in the miRbase, mature nucleotide sequence and reference for their selection.

miRNA	miRBASE	Mature sequence	Reference
**miR-27a-3p**	MI0004746	UUCACAGUGGCUAAGUUCCG	[19]
**miR-146a**	MI0000477	UGAGAACUGAAUUCCAUGGGUU	[20-22]
**miR-103**	MI0000109	AGCAGCAUUGUACAGGGCUAUGA	[22]
**miR-142**	MI0008160	CCCAUAAAGUAGAAAGCACUA	[21, 23]
**miR-93-5p**	MI0000095	CAAAGUGCUGUUCGUGCAGGUAG	[23]
**miR-16**	MI0000070	UAGCAGCACGUAAAUAUUGGCG	[23]
**miR-132**	MI0008156	UAACAGUCUACAGCCAUGGUCGC	[20,22]
**miR-574-3p**	MI0003581	CACGCUCAUGCACACACCCACA	[23]
**cel-miR-39-3p**	MI0000010	UCACCGGGUGUAAAUCAGCUUG	[19, 20]

19. Ioriatti ES, Cirino MLA, Lizarte Neto FS, Velasco TR, Sakamoto AC, Freitas-Lima P, et al. Expression of circulating microRNAs as predictors of diagnosis and surgical outcome in patients with mesial temporal lobe epilepsy with hippocampal sclerosis. Epilepsy Res. **2020**, 166, 106373. DOI: http://dx.doi.org/10.1016/j.eplepsyres.2020.106373
20. Martins-Ferreira R, Chaves J, Carvalho C, Bettencourt A, Chorão R, Freitas J, et al. Circulating microRNAs as potential biomarkers for genetic generalized epilepsies: a three microRNA panel. Eur J Neurol. **2020**, 27,660–666. DOI: http://dx.doi.org/10.1111/ene.14129
21. De Benedittis S, Fortunato F, Cava C, Gallivanone F, Iaccino E, Caligiuri ME, et al. Circulating microRNA: The potential novel diagnostic biomarkers to predict drug resistance in temporal lobe epilepsy, a pilot study. Int J Mol Sci. **2021**, 22, 702. DOI: http://dx.doi.org/10.3390/ijms22020702
22. Zheng P, Bin H, Chen W. Inhibition of microRNA-103a inhibits the activation of astrocytes in hippocampus tissues and improves the pathological injury of neurons of epilepsy rats by regulating BDNF. Cancer Cell Int. **2019,** 19, 109. DOI: http://dx.doi.org/10.1186/s12935-019- 0821-2
23. Brennan GP, Bauer S, Engel T, Jimenez-Mateos EM, Del Gallo F, Hill TDM, et al. Genome-wide microRNA profiling of plasma from three different animal models identifies biomarkers of temporal lobe epilepsy. Neurobiol Dis. **2020**, 144, 105048. DOI: http://dx.doi.org/10.1016/j.nbd.2020.105048

Table S3. Pearson's correlation between microRNA expression data. Statistical significance: *** $p < 0.001$, ** $p < 0.01$, * $p < 0.05$

	miRNA-16	miRNA-27	miRNA-93	miRNA-103	miRNA-132	miRNA-142
**miRNA-27**	0.4226*	-				
**miRNA-93**	0.941***	0.5533**	-			
**miRNA-103**	0.0424	0.0923	0.0408	-		
**miRNA-132**	-0.3506	0.1176	-0.4247*	0.2586	-	
**miRNA-142**	0.7928***	0.6575***	0.8780***	0.0474	-0.1613	-
**miRNA-574**	0.7911***	0.6862***	0.8094***	-0.0609	-0.2448	0.7565***

Table S4. Data from combined miRNAs ROC curve analysis. Statistical significance: *** p < 0.001, ** p < 0.01, * p < 0.05

Diagnostic biomarker performance: control vs epileptic			
**Combined miRNAs**	**AUC**	**p-value**	**Statistical significance**
16-142-93-574	0.9167	0.001257	**
16-142	0.9333	0.000795	***
16-93	0.8667	0.004527	**
16-574	0.875	0.003692	**
142-93	0.9417	0.0006286	***
142-574	0.9417	0.0006286	***
93-574	0.8583	0.00553	**
**142-93-574**	**0.9417**	**0.0006286**	*******
**Prognostic biomarker performance: DSE vs DRE**			
**Combined miRNAs**	**AUC**	**p-value**	**Statistical significance**
16-93-574-132	0.9259	0.00674	**
16-93	0.8704	0.01846	*
16-574	0.8889	0.01336	*
16-132	0.8519	0.02519	*
93-574	0.9074	0.00955	**
93-132	0.9074	0.00955	**
**574-132**	**0.9259**	**0.00674**	******
93-574-132	0.9259	0.00674	**

Table S6. GO pathways in terms of biological process (BP), cellular component (CC) and molecular function (MF) enriched in miR-16, miR-27a-3p and miR-93-5p gene targets, showing the number of genes per pathway (nGenes), the fold enrichment and the FDR of each pathway.

miRNA		Pathway	nGenes	Fold enrichment	FDR
miR-16	BP	Negative regulation of lamellipodium organization	3	62.3	8.4E-03
	BP	Regulation of lamellipodium organization	6	13.8	5.8E-03
	BP	Regulation of protein autophosphorylation	5	12.5	1.4E-02
	BP	Respiratory system development	9	5.1	1.7E-02
	BP	Sodium ion transport	10	5	1.3E-02
	BP	Establishment or maintenance of cell polarity	9	5	1.7E-02
	BP	Regulation of cell development	16	3.8	5.8E-03
	BP	Gland development	13	3.5	1.7E-02
	BP	Regulation of anatomical structure size	15	3.5	1.2E-02
	BP	Regulation of cellular component biogenesis	21	2.5	1.7E-02
	BP	Protein phosphorylation	33	2.4	5.1E-03
	BP	Regulation of organelle organization	24	2.4	1.4E-02
	BP	Peptidyl-amino acid modification	26	2.4	1.3E-02
	BP	Regulation of phosphorylation	25	2.3	1.7E-02
	BP	Positive regulation of transcription, DNA-templated	31	2.3	8.4E-03
	BP	Positive regulation of nucleic acid-templated transcription	31	2.3	8.4E-03
	BP	Positive regulation of RNA biosynthetic process	31	2.2	8.4E-03
	BP	Regulation of phosphate metabolic process	28	2.2	1.7E-02
	BP	Positive regulation of RNA metabolic process	33	2.2	8.4E-03
	CC	Golgi apparatus	30	2.1	5.0E-02
	MF	Amino acid:proton symporter activity	2	62.3	1.8E-02
	MF	GTPase inhibitor activity	3	24.9	1.3E-02
	MF	Amino acid:cation symporter activity	3	20.8	1.8E-02
	MF	Phosphatidylinositol-5-phosphate binding	3	18.7	2.0E-02
	MF	Solute:sodium symporter activity	6	10.1	4.6E-03
	MF	Solute:cation symporter activity	8	9.7	1.1E-03
	MF	Symporter activity	8	6.6	4.6E-03
	MF	Active ion transmembrane transporter activity	9	4.1	1.8E-02
	MF	Secondary active transmembrane transporter activity	8	4.1	2.7E-02

	MF	Protein serine kinase activity	12	4	6.0E-03
	MF	Protein serine/threonine kinase activity	13	3.4	8.9E-03
	MF	Protein kinase activity	17	3.4	4.4E-03
	MF	Phosphotransferase activity, alcohol group as acceptor	17	2.9	8.9E-03
	MF	Kinase activity	19	2.8	6.0E-03
	MF	Transferase activity, transferring phosphorus-containing groups	20	2.5	1.3E-02
	MF	Cytoskeletal protein binding	19	2.3	2.7E-02
	MF	Enzyme regulator activity	23	2.1	1.9E-02
	MF	ATP binding	27	2	1.8E-02
	MF	Adenyl ribonucleotide binding	27	1.9	2.4E-02
	MF	Adenyl nucleotide binding	27	1.9	2.6E-02
miR-27a-3p	BP	Protein targeting to vacuole	5	11.6	1.7E-02
	BP	Associative learning	7	8.2	1.5E-02
	BP	Regulation of circadian rhythm	8	6	1.7E-02
	BP	Multicellular organism growth	9	5.9	1.5E-02
	BP	Lipid homeostasis	10	5.8	1.5E-02
	BP	Regulation of synaptic plasticity	10	4.4	1.8E-02
	BP	Intracellular receptor signaling pathway	17	4.1	4.5E-03
	BP	Striated muscle tissue development	14	3.5	1.7E-02
	BP	Regulation of binding	14	3.5	1.7E-02
	BP	Muscle tissue development	14	3.3	1.8E-02
	BP	Regulation of cellular localization	23	2.5	1.7E-02
	BP	Regulation of cell migration	24	2.4	1.8E-02
	BP	Cell morphogenesis	27	2.3	1.7E-02
	BP	Neuron development	29	2.2	1.7E-02
	BP	Neuron differentiation	34	2.1	1.5E-02
	BP	Generation of neurons	37	2.1	1.5E-02
	BP	Negative regulation of cellular biosynthetic process	37	2	1.7E-02
	BP	Neurogenesis	37	2	1.7E-02
	BP	Positive regulation of macromolecule biosynthetic process	40	1.9	1.7E-02
	MF	Nuclear receptor activity	7	11	1.0E-03
	MF	Ligand-activated transcription factor activity	7	11	1.0E-03
miR-93-5p	BP	Regulation of endocytosis	10	4.8	4.4E-02
	BP	Regulation of vesicle-mediated transport	18	3.3	1.2E-02
	BP	Endocytosis	19	3.2	1.2E-02
	BP	Regulation of transport	38	2.1	1.2E-02
